# Real-world experience with histological confirmation of clinical response of squamous cell carcinoma to topical tirbanibulin

**DOI:** 10.1016/j.jdcr.2023.07.005

**Published:** 2023-07-14

**Authors:** Angela Moore, Kara Hurley, Stephen A. Moore, Luke Moore

**Affiliations:** a Arlington Center for Dermatology, Arlington, Texas; b Arlington Research Center, Arlington, Texas; c Baylor University Medical Center, Dallas, Texas; d Texas Christian University School of Medicine, Fort Worth, Texas; e University of North Texas Health Science Center Texas College of Osteopathic Medicine, Fort Worth, Texas

**Keywords:** clinical research, drug response, general dermatology, medical dermatology, nonmelanoma skin cancer, oncology, squamous cell carcinoma, tirbanibulin, topical

Tirbanibulin 1% ointment is a novel synthetic antiproliferative agent approved by the Food and Drug Administration in 2020 for the treatment of actinic keratoses (AK). Moreover, tirbanibulin has been speculated to have a dual mechanism of action in the inhibition of cell proliferation as follows: (1) binding of tubulin to inhibit microtubule polymerization and (2) binding to the peptide substrate binding site of Src kinase.^1^ We have previously reported the eradication of persistent HPV-57-positive periungual squamous cell carcinoma (SCC) and basal cell carcinoma (BCC) of the ear with tirbanibulin 1% ointment.^2^^,^^3^ Both lesions have shown no evidence of recurrence after 18 months. Elevated Src kinase activity has been established in nonmelanoma skin cancer, with greater activity observed in SCC than BCC.^4^ Consent for the publication of all patient photographs and medical information was provided by the authors at the time of article submission to the journal stating that patient gave consent for their photographs and medical information to be published in print and online and with the understanding that this information may be publicly available.

## Case series

This retrospective case series evaluates the histology after excision of 6 out of 7 biopsy-proven SCC or SCC in situ (SCCIS) after monthly treatment courses of off-label tirbanibulin 1% ointment for 5 consecutive nights. In these cases, topical tirbanibulin was used as adjunctive therapy with the primary initial intent of decreasing lesion thickness and size after biopsy and before surgical excision. The age of the patients in this retrospective case series ranged from 59 to 78 years (mean = 69), with 5 females and 2 males, with all patients being White. Fitzpatrick skin types (FSTs) were II to III, with 4 FST II and 3 FST III. The lesions included 4 SCC and 3 SCCIS, with sizes ranging from 6 to14 mm (mean = 10.1 mm, SD = 3.1 mm). The locations of the lesions were the cheek, temple, scalp, chest, and hand. Patient demographics are presented in Table I. Four patients had lesions that had not decreased in nodularity and size with the use of liquid nitrogen coupled with previous topical therapies. Topical imiquimod had failed in 2 patients, topical fluorouracil had failed in 2 patients, and photodynamic therapy had failed in 1 patient.

**Table I:** Patient demographics and clinical data

Case	Age	Sex	FST	Dx	Location	Size	Failed previous treatment	#Tx courses	Confirmatory excision	Clinical resolution	Histological resolution	Response duration	LSR
1	63	F	II	SCCIS	Cheek	8 mm	None	2	Yes	Yes	Yes	16 months	Moderate
2	59	F	III	SCC	Cheek	6 mm	None	1	Yes	No	Yes	14 months	Mild
3	74	M	II	SCC	Temple	1.4 cm	Imiquimod	3	Yes	No	No	16 months	None
4	78	F	II	SCCIS	Scalp	1.3 cm	Fluorouracil	1	Yes	No	Yes	13 months	None
5	60	F	II	SCC	Chest	1.1 cm	Fluorouracil	1	Yes	No	Yes	16 months	Mild
6	74	F	III	SCC	Hand	1.2 cm	Imiquimod	1	Yes	No	Yes	16 months	Moderate
7	72	M	III	SCCIS	Ear	7 mm	PDT	1	No	Yes	-	16 months	Mild

*Dx*, Diagnosis; *FST*, Fitzpatrick skin type; *PDT*, photodynamic therapy; *SCCIS*, squamous cell carcinoma in situ; *SCC*, squamous cell carcinoma; *Tx*, treatment.

**Fig 1:**
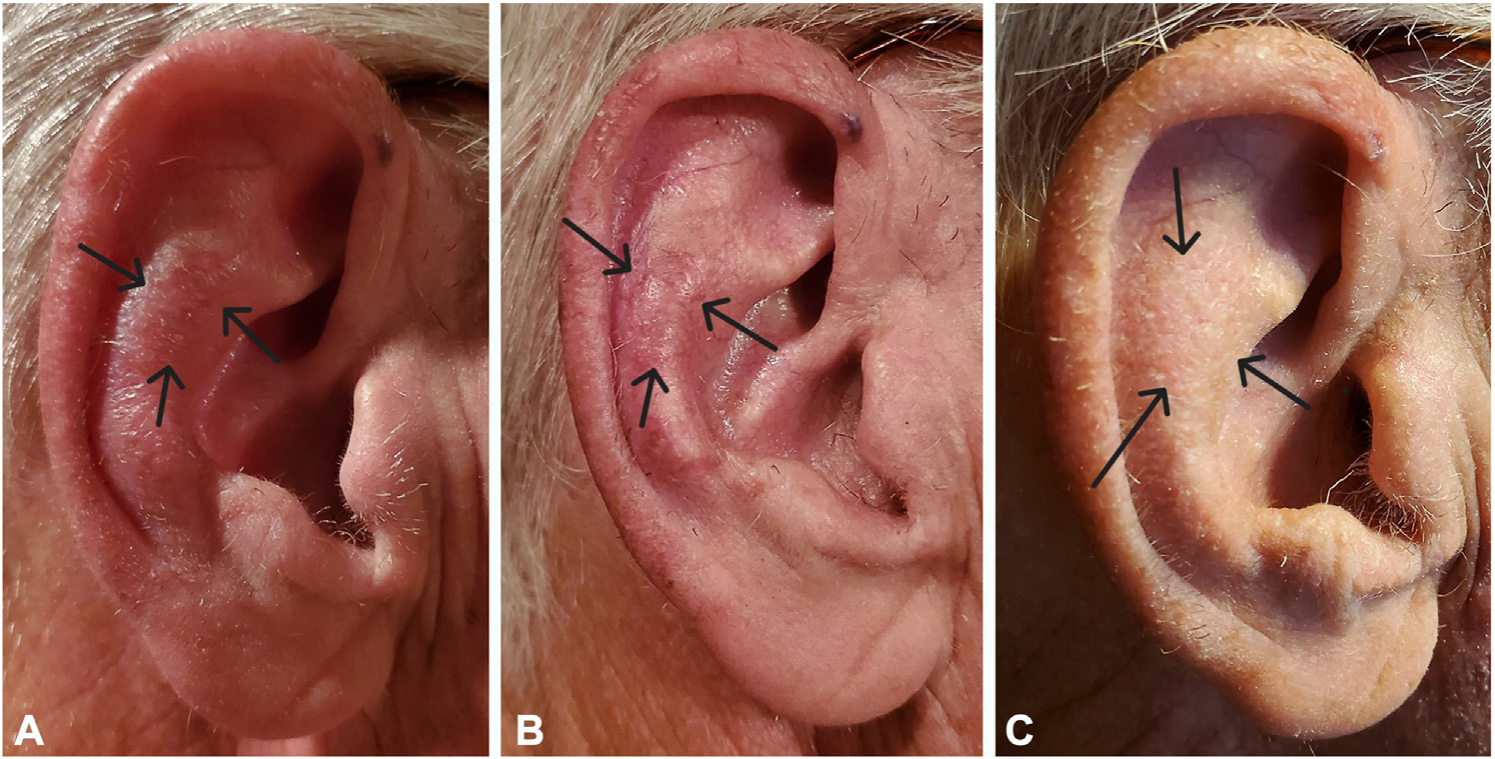
**A,** SCCIS on the right antihelix of a 72-year-old man at baseline, **B,** with mild local skin reaction on day 8 after 5 nights of topical tirbanibulin 1% ointment and **C,** clinical resolution on day 21. *SCCIS*, squamous cell carcinoma in situ.

After the application of liquid nitrogen by the dermatologist on the lesion for a 1-mm halo to increase penetration, patients were instructed to apply a thin layer of tirbanibulin 1% ointment off-label to SCC or SCCIS every night for 5 consecutive nights. A thin layer was achieved by poking a hole in the sachet with a pin and using one-fifth of the sachet per night and storing it in a resealable zipper plastic bag each night. Clinical follow-up was conducted for each patient 1 month after initiating treatment. Surgical excision with 4-mm margins was performed in cases 1-6 (Table I). In case 7, an SCCIS of the right antihelix of the ear was eradicated after 1 treatment course of tirbanibulin 1% ointment with no residual nodularity or erythema (Fig 1). The patient denied surgery as there was no clinical indication.

Histological resolution with no residual cancer cells was confirmed in 5 (83%) of the 6 SCC or SCCIS lesions (Table I) that were excised. Among the 5 lesions that histologically resolved with tirbanibulin, 1 appeared clinically resolved, while 4 appeared clinically improved with decrease in diameter and nodularity but residual erythema. The lesion that clinically resolved also resolved histologically. Among the other 5 lesions with equivocal clinical resolution, 4 were histologically resolved. One to two treatment courses were completed (mean = 1.2, SD = 0.4) in the 5 lesions that resolved histologically, while 3 courses failed to histologically eradicate a 1.4-cm SCC of the temple, but the SCC decreased in diameter and nodularity. The SCCIS of the ear with complete clinical resolution required 1 treatment course. Local skin reactions (LSR) ranged from none in 2 (including the SCC that failed to respond) to mild in 3 and moderate in 2 (Table I). Moderate LSR was defined as obvious erythema, scaling, and/or dryness. Mild LSR or moderate LSR resolved in all patients by day 14. No evidence of recurrence was observed after 13 to 16 months.

## Discussion

Tirbanibulin exhibits a dual mechanism of action, including inhibition of tubulin polymerization and microtubule disruption as well as inhibition of Src kinase signaling.^1^ Src is a serine family kinase, a group of nonreceptor tyrosine kinases involved in angiogenesis and vascular epithelial growth factor.^5^ Tirbanibulin has been shown to inhibit cell growth and migration of triple-negative breast cancer cells. Antitumor effects through the inhibition of Src were demonstrated in both in vitro and in vivo models.^6^ Lee et al established that Src is expressed in all malignant skin cancers, including melanoma, BCC, and SCC. Src activity was reported to be higher in SCC than BCC.4 Additionally, microtubule inhibition has been shown to be integral in the antiproliferative effects of tirbanibulin. Niu et al demonstrated that tirbanibulin binds with very high affinity and specificity to tubulin, halting cell division during the late interphase and triggering apoptosis.^7^

Topical therapies used to treat nonmelanoma skin cancer, such as imiquimod and 5-fluorouracil, are associated with the release of proinflammatory cytokines such as tumor necrosis factor-α and interleukin-8 (IL-8). These proinflammatory cytokines are believed to mediate moderate-to-severe LSR often associated with topical imiquimod and 5-fluorouracil.^8^^,^^9^ Preclinical results from the ATNXUS-KX01-001 study demonstrated that incubation with tirbanibulin for 24 hours induced only a small increase in IL-8 at the highest dose compared to 5-FU that induced a significant increase in tumor necrosis factor-α and IL-8. Investigators have suggested that the mild proinflammatory response of tirbanibulin may be associated with milder LSR.^10^ In pooled phase 3 trials of tirbanibulin for the treatment of AKs, LSR included mild-to-moderate erythema, desquamation, application-site pruritus, and application-site pain.^11^ Post hoc pooled analysis of data from 2 phase 3, randomized, double-blinded, vehicle-controlled studies of tirbanibulin 1% ointment for the treatment of AKs demonstrated that clearance was not dependent on LSR.^12^ Among our cases, 2 patients experienced no LSR, 2 patients experienced mild LSR, and 2 patients experienced moderate LSR. None of the patients experienced severe LSR, which would have included painful erythema, desquamation, hypopigmentation, and/or hyperpigmentation.

This study demonstrates histological clearance of SCC and SCCIS with tirbanibulin 1% ointment in 5 (83%) of 6 lesions, even in cases where clinical resolution was equivocal with persistent erythema. These findings suggest that SCC and SCCIS with equivocal clinical response to topical tirbanibulin may benefit from biopsy of the residual site of erythema and may not warrant immediate excision with standard 4-mm margins. Complete clinical response may not warrant biopsy or excision, as demonstrated in case 7 with sustained resolution after 18 months. These findings are consistent with previous findings on the use of topical imiquimod; complete clinical response correlates with histological response, but clinical improvement with residual erythema may or may not correlate with histological clearance. Cases with higher LSR in this series were histologically resolved, but confirmation of histological resolution could be done even with mild or no LSR and equivocal clinical resolution. These findings suggest that judicious application of topical tirbanibulin may still generate clinical response and efficacy in adjunctive treatment of SCC and SCCIS with topical tirbanibulin, despite low LSR and the fact that equivocal clinical resolution may still correlate with histological clearance. Cases with equivocal resolution may need biopsy rather than full excision, while the cases with clinical resolution do not need biopsy. Of note, absence of residual cancer on excision following biopsy is possible. In this case series, 6 of 7 SCC or SCCIS lesions were eradicated with tirbanibulin 1% ointment after biopsy, even in cases where clinical resolution was equivocal with persistent erythema. Based on these findings, larger prospective studies evaluating the efficacy of topical tirbanibulin for the treatment of SCCIS and as an adjunctive treatment of SCC are needed.

## Conflicts of interest

Angela Yen Moore, MD has received honoraria and/or research funds from Almirall, LLC. None of the other authors have any conflicts of interest to declare.
